# The accuracy of edentulous arch impression between intraoral scanner and laboratory scanner: a scoping review

**DOI:** 10.1038/s41405-025-00300-4

**Published:** 2025-02-13

**Authors:** Athiyyah Aura Achmadi, Rasmi Rikmasari, Fahmi Oscandar, Vita Mulya Passa Novianti

**Affiliations:** 1 Department of Prosthodontics, Faculty of Dentistry, Universitas Padjadjaran, Bandung, Indonesia; 2 Department of Oral and Maxillofacial Radiology, Faculty of Dentistry, Universitas Padjadjaran, Bandung, Indonesia

**Keywords:** Removable prosthodontics, Fixed prosthodontics, Removable prosthodontics

## Abstract

**Objective:**

This study aims to compare the utilization of intraoral scanners and laboratory scanners as an alternative impression method in fully and partially edentulous cases.

**Materials and methods:**

This scoping review that implemented the PRISMA-ScR instrument and the methodological approach by Arksey and O’Malley. A comprehensive search was conducted across four databases (PubMed, Scopus, SpringerLink, and ScienceDirect) to retrieve articles published within the last decade. Inclusion criteria were established to identify articles that analyzed the accuracy of both intraoral scanners and laboratory scanners in edentulous cases. Data extraction was performed and results were presented in tables. Subsequently, a thematic analysis was conducted to conclude the accuracy of the intraoral scanners and laboratory scanners in edentulous cases.

**Results:**

A total of 312 articles were retrieved from four databases. After eliminating duplicates and screening based on titles, abstracts, and eligibility criteria, eight articles were selected for detailed analysis of the accuracy of each technology. Most studies investigated fully and partially edentulous arches and demonstrated the utilization of intraoral scanners and laboratory scanners. Some studies additionally analyzed the correlation between various factors influencing digital scans and the condition of edentulous arch. Evaluations of edentulous digital impressions have been conducted, with an assessment of the reliability of intraoral scanners and laboratory scanners.

**Conclusion:**

Intraoral scanners are extensively utilized and demonstrate considerable promise for edentulous impression procedures. However, morphological differences may impact scanning outcomes.

## Introduction

Edentulism is divided into fully edentulous and partially edentulous cases. Generally, clinical practitioners manage edentulous cases by performing complete or partial denture procedures. Based on the previous study, Tambe et al. [1] and Zarb et al. [2] mentioned that the effectiveness of denture treatment depends on how well the impression captures all the necessary surfaces and tissues to achieve optimal retention, stabilization, and support factors. Another previous study by Jain et al. [3], emphasized that the difference in resiliency between supporting tissues can cause instability in the denture. The challenge is to balance the resiliency between the less resilient periodontal ligament of the abutment teeth and the more resilient mucosa of the gingiva, ensuring that the partial denture functions correctly. Various tissues in partially edentulous cases require a reasonable denture base adjustment. In edentulous cases, it is essential to accurately capture the ridge area, as this area will transmit chewing loads from the prosthesis to the tissues. Therefore, creating an accurate impression to produce a prosthesis that can reduce retention and stabilization problems while improving masticatory function is crucial. The integration of digital dentistry is expected to initiate a significant advancement in assessing the condition of the patient’s oral cavity.

The development of digital technology resulting from advancements in science and technology has profoundly influenced the field of dentistry. Nowadays, digital impressions or optical scanning are replacing conventional impressions [4, 5]. Digital impressions utilizing computer-aided design (CAD) and computer-aided manufacturing (CAM) technology are acquired via direct or indirect scanning [6–8]. Direct scanning employs an optical record system, augmented by an intraoral scanner (IOS), to capture the oral cavity’s condition directly [9, 10]. A study by Goodacre et al. [4] stated that IOS can acquire a 3D color display, which enables visualization on a computer monitor effectively. Natsubori et al. [11] also explained that the indirect method initiates the fabrication of conventional impression and cast models, followed by digitization through scanning with a laboratory scanner. The acquired data were imported into the standard triangle language (STL) file format utilizing the applied software [11, 12]. Another study conducted by Vafaee et al. [13] concluded that enhancing the accuracy of dental technology is essential for minimizing the shortcomings of traditional approaches to impression-taking and fabrication. However, Fang et al. [9] and Schmalzl et al. [14] identified that achieving accuracy in digital impressions on the edentulous arch presents a challenge due to the smooth surface needing more distinct features, thus complicating the processes of tracing and stitching. When it comes to scanning dental structures, it has been mentioned in the previous study by Schmalzl et al. [14] that the accuracy decreases as more teeth are scanned using IOS. Previous studies have highlighted significant advancements in digital dentistry. However, significant attention is needed to understand the effectiveness of IOS and laboratory scanners for edentulous impressions. These scanners are crucial in capturing detailed anatomical features for prosthetic accuracy. Furthermore, thoroughly examining their capabilities, including challenges related to scanning specific anatomical structures and software-related issues, is critical for refining future studies and enhancing clinical applications of digital scanning technologies in prosthetic treatment. Nevertheless, the accuracy exhibited by both the IOS and laboratory scanners remained within clinically acceptable parameters.

The authors have comprehensively reviewed existing studies, encompassing both in vivo and in vitro analysis of IOS and laboratory scanners. The authors also observed insufficient literature reviews regarding the accuracy of edentulous arch impressions between both scanners. This reinforces the authors’ decision to focus their study through a scoping review to compare the utilization of intraoral scanners and laboratory scanners as an alternative impression method in fully and partially edentulous cases.

## Material and methods

This study employed a scoping review utilizing the Preferred Reporting Items for Systematic Reviews and Meta-Analyses extension for Scoping Review (PRISMA-ScR), following the methodological approach established by Arksey and O’Malley [15]. This scoping review utilizes various tools and supporting materials, including a laptop and smartphone, Microsoft Word and Excel (Microsoft Corp., Redmond, WA, USA), Mendeley Reference Manager, internet networks, and databases (PubMed, Scopus, SpringerLink, and ScienceDirect).

### Inclusion and exclusion criteria

The included articles were published within the last ten years (2013–2023) and examined the accuracy of edentulous arch impressions utilizing IOS and laboratory scanners through in vivo or in vitro analysis. These included articles focused on digital impressions for edentulous arches without implants. Articles must be published in English and accessible in full-text internationally. The excluded articles were traditional reviews or case reports, solely focusing on IOS or laboratory scanners and addressing CBCT scans.

### Search strategy

This study was conducted from February to May 2024, involving a literature search across four databases. The article search strategy utilized Boolean Operators, explicitly combining the words “AND,” “OR,” and “NOT.” The search was conducted using the following keywords: (accuracy) AND (digital impression) OR (“intraoral scanners”) AND (extraoral scanners) OR (“laboratory scanners”) OR (3D Scan) AND (edentulous). This scoping review adopts the PCC (Population, Concept, and Context) framework. The target population includes cast models or individuals with fully or partially edentulous maxilla and/or mandible. The primary concept of this review is to assess the accuracy of digital impressions obtained from IOS and laboratory scanners. The context addressed in this study includes factors influencing impression accuracy, such as impression technique, tissue structure morphology, and technology performance. After conducting the search, articles were screened based on their titles, abstracts, and eligibility criteria. Subsequently, data from the included articles will be extracted and presented in a table, followed by a thematic analysis. Thematic analysis is a practical approach to exploring qualitative data and comprehending the perceived phenomenon from the researcher’s perspective [16].

### Risk of bias assessment and quality appraisal

As this study was limited to a scoping review, it did not require statistical analysis. Nonetheless, we performed a quality assessment for each study using the Joanna Briggs Institute (JBI) Critical Appraisal Tools to assess risk of bias [17].

## Results

**Fig. 1:**
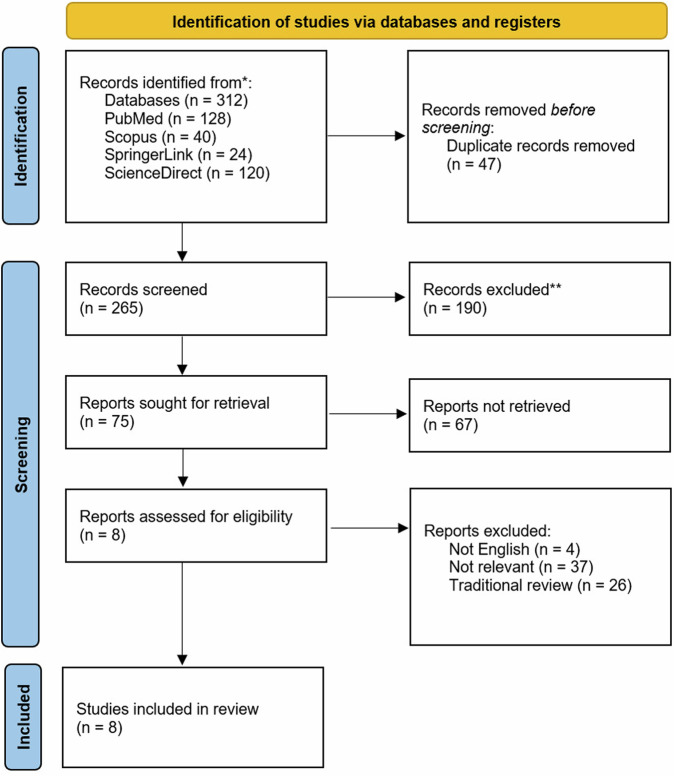
The PRISMA flow diagram for selecting studies. The PRISMA flowchart illustrates the process of literature search, selection, and data analysis.

**Fig. 2:**
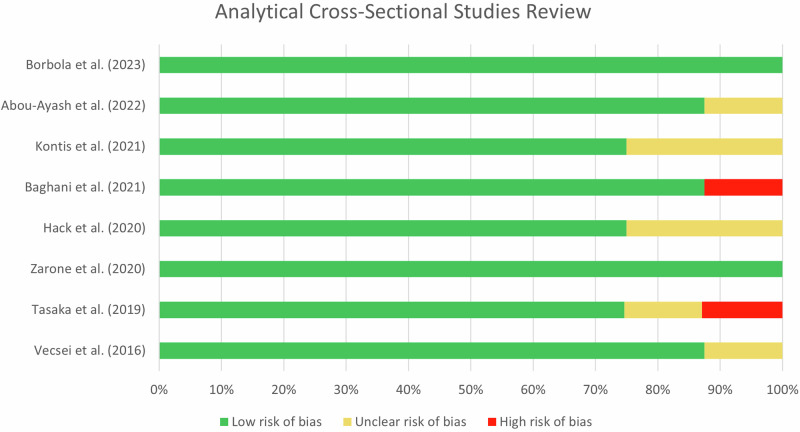
Risk of bias analytical cross-sectional studies. The bar chart illustrates the percentage of bias identified in each study, categorizing the studies by low, moderate, or high risk of bias.

The searches through four databases (PubMed, Scopus, SpringerLink, and ScienceDirect) yielded 312 articles. Duplicate articles were removed through Microsoft Excel, resulting in 265 articles. Screening based on titles and abstracts resulted in 75 articles. The following selection involved full-text screening to assess eligibility criteria, resulting in eight articles for further analysis (Table 1). The article selection process in this study is illustrated in the PRISMA-ScR diagram (Fig. 1). All studies employed an analytical cross-sectional study design. Additionally, the quality assessment and potential bias risk of the analytical cross-sectional studies are presented in Fig. 2. All studies exhibited low risk of bias, suggesting they possess high qualities.

**Table 1:** The data extraction for included studies.

Author (Year)	Aims	Intraoral Scanner	Laboratory Scanner	Sample	Methods	Findings
Vecsei et al. [18] (2016)	Comparing the scanning accuracy of direct and indirect methods.	iTero, Trios, Cerec Omnicam	Straumann CARES Scan CS2 Visual 8.0	Partially edentulous maxillary PMMA model	- The model was directly scanned by three IOSs. - PVS impression and a plaster model were scanned by a LBS. - Data in STL format were compared to calculate deviations and determine accuracy.	- Both methods’ accuracy is influenced by the arch’s length. - The direct method is consistent in scanning curves at varying distances. - The indirect method is more accurate in full-arch scanning.
Tasaka et al. [19] (2019)	Evaluating the accuracy of IOS for scanning edentulous residual ridges.	Trios 2	D900	Full edentulous maxillary model and bilateral free end edentulous mandibular model	- The simulation models were scanned by a LBS to acquire reference data. - The model was scanned five times by an IOS. - CAD software uses a double scan technique to superimpose IOS data with reference data.	- Both IOS and LBS still provide satisfactory accuracy for edentulous cases. - Soft tissue structures may impact the accuracy of IOS.
Zarone et al. [20] (2020)	Comparing accuracy for edentulous maxilla between IOS and LBS.	Trios 3	DScan 3	Full edentulous maxillary typodont model	- The models were scanned with an IOS. - Ten plaster models of polysulfide impressions were digitized utilizing a LBS. - STL files were transferred into the software to calculate the accuracy values in micrometer (µm).	In digital denture manufacturing, IOS exhibits superior trueness and precision compared to LBS.
Hack et al. [25] (2020)	Assessing the accuracy of digital optical impressions in vivo for edentulous arch.	Lava Chairside Oral Scanner (C.O.S)	D700	Full edentulous maxilla and/or mandible	- The PVS impressions underwent three scans utilizing a LBS and an IOS. - The type III plaster model of the PVS impression underwent three scans utilizing a LBS. - All data were loaded into the 3D evaluation software for deviation assessment.	Although IOS and LBS are currently unable to scan mobile tissues, these scanners can still be effectively utilized for scanning the edentulous arch.
Baghani et al. [21] (2021)	Evaluating the accuracy of an entire arch with three IOSs and one LBS, in comparison to an industrial 3D scanner.	Trios 3, Cerec OmniCam Carestream CS 3600	Deluxe desktop scanner	Partially edentulous maxillary typodont model	- The reference model underwent scanning with three IOS and one LBS. - All STL files were imported into the software.	- The presence of edentulous areas significantly impacts the performance and accuracy of both IOS and LBS. - The accuracy of the Carestream ranks the lowest among all the IOS tested.
Kontis et al. [22] (2021)	Comparing IOS accuracy with LBS on edentulous maxillary model impressions.	Cerec Primescan and Cerec Omnicam	In EOS X5	Full edentulous maxillary model	- The PEEK model was scanned using two IOSs. - Twenty-five PVS impressions and type IV plaster were scanned with a LBS. - Data were transferred into the analysis software.	- Primescan shows excellent trueness, followed by In EOS X5 results. - In EOS X5 demonstrates superior precision.
Abou-Ayash et al. [23] (2022)	Evaluate the accuracy and time effectiveness of the IOS and LBS in three partially edentulous cases.	Primescan and Trios 3	S600 Arti	Three partially edentulous maxillary models (Model 1: Kennedy Class IV; Model 2: Kennedy Class II FDI #13 and #16; Model 3: Kennedy Class II FDI #14 and #16)	- Direct method: ten trial scans were performed using two IOSs. - Indirect method: fourteen vinylsiloxanether impressions were made, filled with type IV plaster, and scanned using a LBS. - All data are exported in STL format and brought into CAD software.	- Direct scanning demonstrates comparable or superior accuracy compared to indirect scanning. - Primescan exhibits higher accuracy, particularly on model 3, and is more efficient than LBS.
Borbola et al. [24] (2023)	- Compare the precision of five LBS and one IOS. - Compare the trueness of the IOS measure by LBSs and an industrial scanner.	Medit i700	Ceramill Map 400, Medit T710, PlanScan Lab, CS Neo, 3Shape E4	Three partially edentulous maxillary models and one dentate model	- Models were scanned with five desktop scanners and one IOS, following the manufacturer’s protocol. - Scan results are exported in STL format and transferred into a comprehensive metrology program. - Trueness and precision are calculated from the scanning outcomes.	- The Medit i700 demonstrates exceptional accuracy for full-arch scans. - Both the 3Shape E4 and Medit T710 demonstrate superior accuracy compared to other LBS systems. - LBS provides precise capabilities for prosthetic workflows.

*IOS* intraoral scanner, *LBS* laboratory scanner, *STL* standard triangle language, *CAD* computer-aided design, *3D* three-dimensional, *PVS* polyvinyl siloxane, *PEEK* polyetheretherketone, *PMMA* polimethylmethacrilic acid, *FDI* Fédération Dentaire Internationale.

### Included studies characteristics

The majority of included studies were published between 2016 and 2023. This qualitative synthesis comprised seven in vitro studies [18–24] and one in vivo study [25]. Among these, one study examined fully and partially edentulous cases [19], three studies focused solely on fully edentulous cases [20, 22, 25], and four included only partially edentulous cases [18, 21, 23, 24]. The IOS utilized in these studies varied, with the Trios (3Shape Headquarters, Copenhagen, Denmark) being the most common, featured in five studies [18–21, 23]. The Cerec Omnicam (Dentsply Sirona, Bensheim, Germany) was utilized in three studies [18, 21, 22], and Cerec Primescan (Dentsply Sirona, Bensheim, Germany) in two studies [22, 23]. Other IOS used included the iTero (Align Technology B.V., Amsterdam, Netherlands) [18], Lava Chairside Oral Scanner (3M ESPE, St. Paul, MN, USA) [25], and Medit i700 (Medit, Seoul, South Korea) [24]. The laboratory scanner predominantly utilized was the Medit T710 (Medit, Seoul, South Korea) [24]. Other laboratory scanners included the Straumann CARES Scan CS2 (Institut Straumann AG, Basel, Switzerland) [18], D900 (3Shape A/S, Copenhagen, Denmark) [19], DScan 3 (EGS, Bologna, Italy) [20], D700 (3shape, Copenhagen, Denmark) [25], Carestream CS3600 (Carestream Dental, Rochester, USA) [21], In EOS X5 (Sirona Dental Systems, Bensheim, Germany) [22], S600 Arti (Zirkonzahn GmbH, Gais, Italy) [23]. Another study included the 3Shape E4 (3Shape A/S, Copenhagen, Denmark), Ceramill Map 400 (Amman Girrbach AG, Koblach, Austria), CS Neo (Cadstar Technology GmbH, Bischofshofen, Austria), and Planscan Lab (Planmeca, Helsinki, Finland) [24]. Most studies complied to the scanning protocols recommended by the manufacturers. All studies analyzed deviation analysis by superimposing scan data from IOS and laboratory scanner using CAD software. Statistical analyses were performed to assess the trueness and precision values.

### Fully edentulous

A study by Tasaka et al. [19] focused on the accuracy of IOS in a fully edentulous maxilla by comparing the scan data to reference data from a laboratory scanner. The findings indicated that the accuracy is clinically acceptable, though the operator’s technique can influence it [19]. Similarly, Zarone et al. [20] compared an IOS with a laboratory scanner for a fully edentulous maxilla using a reference typodont. The results substantiated that direct scanning with the IOS produced higher accuracy compared to laboratory scans via polysulfide impressions and stone casts [20]. However, when using the laboratory scanners, there were no significant differences in trueness and precision between polysulfide impressions and stone cast scans [20]. Hack et al. [25] encountered that the highest deviations (≥500 μm) were identified in the peripheral seal zone. Kontis et al. [22] discovered that the IOS exhibited the highest trueness in both linear and angular parameters; this means that direct scanning provided results closest to the actual dimensions of the edentulous arch. In this study, the highest precision was observed with the laboratory scanner [22].

### Partially edentulous

The previous study conducted by Vecsei et al. [18] emphasized that the condition of the arch, the presence of edentulous areas, and the distance between abutments can impact scanning accuracy. Specifically, the reduced number of reference points in edentulous alveolar crests affects the accuracy [18]. Tasaka et al. [19] reported significant differences in accuracy for partially edentulous, particularly in the premolar and midline areas. This study also noted the consistent accuracy across different operators [19]. Baghani et al. [21] also discovered that the accuracy of IOS and laboratory scanners is influenced by the scanned tissue characteristics and anatomical irregularities. Abou-Ayash et al. [23] explained that IOS exhibited the lowest deviations compared to laboratory scanners and required the least time for scanning and processing procedures. Borbola et al. [24] also indicated that IOS showed sufficient accuracy for complete arch scans in partial edentulous cases, although its precision was lower than the best laboratory desktop scanners.

## Discussion

The outcome of an edentulous treatment relies on the quality of the impression stage. Phoenix et al. [26] emphasized the importance of prosthetic accuracy in ensuring denture adaptation to preserve both the remaining teeth and the supporting mucosa. Accuracy measurements, in accordance with the International Organization for Standardization (ISO) 5725-1, are evaluated based on two aspects, including trueness and precision [27]. Trueness describes how closely the measurement results match the actual dimensions of the measured reference object [28]. Precision signifies the proximity of measurement results obtained from repeated measurements [29, 30]. In line with Çakmak et al. [31], it was identified the importance of high scanner accuracy in achieving optimal scanning outcomes and accurately reproducing the dimensions of the scanned object.

Study results indicate that various scanning devices, including intraoral scanners (IOS) and laboratory scanners, have advantages and disadvantages regarding their precision and trueness. The majority of eight articles reported that scanning with IOS showed more accurate results than scanning with a laboratory scanner, especially regarding trueness [18–25]. This aligns with Abou-Ayash et al. [23], who confirmed that the trueness and precision of IOS are nearly equivalent to or better than those of laboratory scanners. This observation corresponds with Natsubori et al. [11], who explained that IOS has a smaller camera size and the capability to capture data within a smaller area per scan, resulting in higher accuracy. This performance of IOS is advantageous for clinical workflows requiring detailed anatomical impressions with minimal patient discomfort. This capability enhances prosthodontic outcomes by allowing accurate, real-time visualization of the scanned areas. However, the evidence from these studies does not rule out the critical role of laboratory scanners in digital impressions. Kontis et al. [22] determined that laboratory scanners have superior precision results compared to IOS. The superior precision of laboratory scanners is possible because measurements are carried out in stable and controlled conditions without external light interference [19, 32]. Additionally, laboratory scanners can capture models with a broader area, reducing the possibility of errors when combining images [22, 33, 34]. This could also occur due to repeatedly superimposing a large amount of data, leading to the potential for IOS to produce less precise outcomes [22]. A study by Natsubori et al. [11] and Lee et al. [35] highlighted that the laboratory scanner’s support table device has the capability to move in all directions, and several cameras can facilitate scanning from various angles across expansive areas. This is in accordance with Vecsei et al. [18], who stated that laboratory scanners can provide more precise results for fully edentulous arch cases. Their ability to minimize errors through stable environments and sophisticated image-stitching processes makes them indispensable for extensive data-stitching cases. These differences underscore the importance of selecting the appropriate scanner based on specific clinical needs.

Most of the eight studies utilized various types of IOS and laboratory scanners, significantly influencing the trueness and precision of digital scanning in edentulous arches [36]. Schmalzl et al. [14] highlighted Trios for its widespread recognition and high accuracy. Similar results were conducted by Kontis et al. [22] and Schimmel et al. [37], that Primescan and Trios demonstrate high trueness in scanning full edentulous models, making them suitable for denture fabrication. Another type of IOS, as referred to by Borbola et al. [24], is the Medit i700, known for its optimal accuracy in scanning partially edentulous arches. Furthermore, Trios, Carestream CS3600, and Omnicam have shown high accuracy in scanning the labial region of the maxillary anterior teeth [38]. A study conducted by Abou-Ayash et al. [23] also asserted that Primescan demonstrates greater accuracy than Trios 3, Trios 4, and S600 Arti for scanning both maxillary and mandibular areas. Similar results were reported by Fattouh et al. [39], which outperforms Trios 3 in accuracy due to its ability to reach challenging areas for scanning. Additionally, Primescan utilizes a video-based scanning technology, enabling it to capture thousands of images quickly, whereas Trios 3 relies solely on photo-based devices [39]. Nevertheless, all these types of IOS can still facilitate scanning in edentulous cases.

The trueness of IOS may vary depending on its version and software [31]. This observation is consistent with Winkler et al. [40], who stated that advancements in scanner systems tend to reduce weaknesses in previous versions. Schmalzl et al. [14] also made a similar statement that older scanner systems are generally less precise than newer ones, suggesting that technological advancements may improve accuracy. Winkler and Gkantidis also alluded that hardware and software advancements contribute to minimizing minor inaccuracies [40]. Therefore, ongoing study in this area remains essential. Schmalzl et al. [14] also conducted tests on two generations of Trios, Trios 3 and Trios 4, across four software versions. Their study indicates that appropriate software updates can enable the previous generation (Trios 3) to match the performance of the newer generation (Trios 4) in full-arch cases [14]. This update will enhance the stitching mechanism and reduce the distortion effect on the alveolar ridge. While the structure of the edentulous arch may still influence its accuracy, software updates appear capable of generating more precise STL files [14]. Despite variations in trueness and precision among different types of IOS, all demonstrate favorable performance in accurately representing the entire anatomy of the full-arch.

The types of laboratory scanners used in these eight articles vary considerably. Notably, one study utilized the Medit T710 scanner [24]. Borbola et al. [24] concluded that the Medit T710 and 3Shape E4 laboratory scanners demonstrate high accuracy and may be utilized as reference data for evaluating IOS accuracy. Other laboratory scanners, such as the 3Shape E4, Ceramill Map 400, Planscan Lab, and CS Neo, demonstrate satisfactory accuracy in fabricating full-arch prostheses [24]. A comparison of accuracy among various laboratory scanners indicates that the detected disparities are not clinically significant [20]. A previous study indicates that variations in deviation among scanner types may result from potential errors in data merging and accumulation software during processing [41].

Based on the analysis of these eight articles, it is crucial to acknowledge that several factors significantly impact the accuracy of both scanning methods. Direct scanning is mainly affected by saliva, crevicular fluid, blood, patient’s breathing and movement, gag reflex, and movement of the tongue, lips, cheeks, and the scanning procedure itself [18, 21, 31]. Shah et al. [42] highlighted inaccuracies from impression material and cast model dimensions, as well as operator-related influence, and the accuracy of indirect scanning. This observation aligned with Çakmak et al. [31] and Ke et al. [32], who emphasized that the operator’s clinical expertise directly affects the outcome of conventional impression and may lead to impression deformation. Moreover, Müller et al. [43] explained that the accuracy of IOS scanning depends on the operator’s hand movement skills. Thomas et al. [44] also observed that less experienced operators typically require more time to conduct a scan than their highly experienced counterparts.

Most of the reviewed articles involve conventional impression procedures, highlighting the influence of the impression process on the scanning results obtained by the scanner. Zarone et al. [20], Ke et al. [32], and Rhee et al. [41] asserted several factors that could influence accuracy during the conventional impression process. These factors include deformation of the impression material, expansion, shrinkage, and potential release from the stock tray [20, 32, 41]. This observation is consistent with Noort’s research, which indicates that impression materials susceptible to distortion, whether during storage or before being poured with plaster, can significantly compromise the final result’s quality [45]. A similar statement was made by Rhee et al. [41], who confirmed that dimensional changes in conventional impression significantly influence the accuracy of scanning outcomes obtained by laboratory scanners. These factors define why IOS scanning regularly achieves greater accuracy than scanning with a laboratory scanner, necessitating a cast model as the scanning target.

Another study by Emam et al. [46] explained that substrate characteristics, such as optical properties and surface roughness, critically affect scan quality. Four reviewed articles focused on scanning plaster models using laboratory scanners [18, 22, 23, 25]. Vandeweghe et al. [47] also remarked that the rough surfaces of plaster models significantly lead to more significant challenges during scanning. Similarly, Sason et al. [8] noted that characteristics of plaster models, such as translucency, matte surfaces, and porosity, scatter light diffusely. This scattering reduces the intensity of focused light, potentially leading to errors in the scanning process [8]. Meanwhile, the other two studies utilized the typodont model [20, 21]. Zarone et al. [20] encountered that digital scanning of typodont models revealed greater accuracy than conventional impressions and plaster models scanned with laboratory scanners. This superiority can be attributed to the lack of distortion in typodont models [20]. Kontis et al. [22] also utilized the polyetheretherketone (PEEK) model, known for its non-reflective surface. The material characteristics of the scanned models significantly enhanced the scanning quality demonstrated in their study.

Most studies have focused on evaluating the accuracy of IOS and laboratory scanners for fully and partially edentulous arches. Variations in the structure and morphology of fully and partially edentulous arches significantly influence the scanner’s ability to capture details accurately. For instance, fully edentulous cases may present different scanning challenges than partially edentulous cases due to the absence of teeth as reference points. Vecsei et al. [18] reported a similar significant challenge in the digital impression of edentulous arches related to image stitching. Similar challenges were also noted by Tasaka et al. [19], Deferm et al. [48], and Gan et al. [49], that difficulties in tracing palatal morphology and residual ridges (free-end saddles), as well as dealing with tori palatini, which can affect trueness and lead to stitching errors. Moreover, compared to scans of tooth structure, obtaining accurate assessments of edentulous arches proves to be quite challenging for the IOS system and its software [21, 22, 25]. A study by Ke et al. [32] established that accurate digital impressions with minimal errors were achieved by placing landmarks on an edentulous model. This evidence is consistent with Vecsei et al. [18], who discovered that the more structures identified by the scanner system, the more influential the performance of the image stitching process. Tasaka et al. [19] also mentioned that optical impressions can effectively be utilized for residual ridges in edentulous areas with careful consideration of these challenges. Understanding these factors is crucial for optimizing scanning protocols and ensuring accurate clinical outcomes.

The edentulous arch includes areas of mobile tissues, including the sublingual and vestibule regions, characterized by smooth surfaces and coated with saliva [19, 25]. These regions are critical for denture retention [25]. It is noted that current IOS systems still need to fully achieve the capability to produce functional impressions with border molding [22]. However, Tasaka et al. [19] explained that IOS can still be utilized in partially edentulous cases to create accurate anatomy impressions of the remaining tooth structures and mucous membranes. Laboratory scanners rely on static impressions made with materials designed to capture the functional shape of the vestibule during border molding, ensuring an accurate impression of this region under controlled conditions. However, their accuracy in capturing the vestibule depends significantly on the quality of the initial impression, as errors in the deformation of the impression material can compromise results [20, 41]. While IOS offers advantages in efficiency and convenience, traditional impressions are still needed to improve accuracy in fully edentulous cases. This comparison emphasizes the importance of selecting the appropriate scanning method based on clinical goals and the specific anatomical challenges of edentulous patients. Furthermore, previous studies by Lo Russo et al. [50] and Baba et al. [51] also noted that the suitability of dentures manufactured with CAM technology, with superior scanner accuracy, can establish intimate contact between the intaglio surface of the denture and the underlying soft tissue. Therefore, these advancements can improve denture retention and overcome some disadvantages.

Soft tissue displacement profoundly affects the accuracy of the denture border [20]. A study by Zarone et al. [20] mentioned the tissue displacement in the buccal vestibule and alveolar crest. Hack et al. [25] similarly reported challenges in scanning the tuberosity and vestibular tissue in the maxilla using IOS. Furthermore, the mandible presents difficulties in scanning areas such as the retromolar pad, vestibule, sublingual area, and mobile tissue. Additionally, analysis of the complete edentulous maxilla and mandible indicates that the attached gingiva exhibits the lowest deviation [25]. In contrast, the maxilla demonstrates the highest deviation in the soft palate and vestibule, while the mandible shows prominent discrepancies in the sublingual and vestibule areas [25]. IOS often fails to capture mobile tissue effectively, excluding these areas from scanning. Therefore, minimal deviation occurs in attached gingiva scanning, as IOS primarily focuses on hard tissue and stable areas. Additionally, Baghani et al. [21] identified the most significant discrepancies in scanning interdental spaces using laboratory scanners. This outcome corresponds with Yatmaz et al. [52], who emphasized the challenges of capturing interproximal and narrow areas with such scanners. Vecsei et al. [18] stated that IOS performs well only in accessible areas.

The length of the arch significantly influences the accuracy of both IOS and laboratory scanners [18]. Based on the previous study by Nedelcu et al. [38], longer scanned areas increase the complexity of the stitching process, thereby enhancing the potential for errors. According to the earlier study, Vecsei et al. [18] found that scanning accuracy improves at shorter distances. Laboratory scanners achieve higher trueness at medium distances, although their precision may be lower than IOS systems [18]. Flügge et al. [53] also highlighted the superior precision of laboratory scanners in full-arch cases compared to IOS. However, Vecsei et al. [18] concluded that IOS consistently demonstrated reliable scanning performance across any distance compared to laboratory scanners.

Müller et al. [43] concluded that the scanning strategy significantly impacts accuracy. Another study conducted by Zarone et al. [54] also confirmed that scanning from occlusal, buccal, and palatal aspects showed the most accurate results in edentulous maxillary arches, mainly when rugae and ridge morphology are clearly defined. This study corroborates Jamjoom et al. [36], who identified that initiating the scanning procedure posteriorly, followed by lingual or palatal scanning, returning to the occlusal surface, and scanning the buccal side, provided optimal accuracy for full edentulous arches. Sequential scanning methods were noted to enhance anatomical detail and avoid distortion during stitching. Ender et al. [55] recommended rotating the intraoral scanner head by 45° when transitioning from the occlusal to the buccal or lingual surface to reduce stitching errors in intraoral scanning.

Several types of IOS used in related studies, such as Trios, Omnicam, iTero, and Carestream, are demonstrated for their ability to scan without the application of scanning powder [18, 56, 57]. However, Hack et al. [25] conducted an in vivo study using the Lava COS, which requires a small amount of powder for scanning the edentulous maxilla and mandible. The study also mentioned that saliva and tongue movement may disrupt the proper powder application, requiring its reapplication during scanning [25]. Furthermore, a study by Abou-Ayash et al. [23] and Rhee et al. [41] indicated that nonuniform powder application can compromise scanning accuracy. Therefore, opting for a powder-free system enhances user comfort during scanning [41].

Based on the comprehensive review of multiple studies, IOS is the optimal choice for edentulous arch impressions due to its advantages. Its accuracy ensures precise capture of detailed anatomical edentulous features and enhances clinical workflow efficiency. Furthermore, IOS reduces the time required for impression-taking, which allows for comprehensive assessment and planning, ultimately leading to superior prosthodontic treatment outcomes. This study thoroughly explores the potential of IOS and laboratory scanners in fully and partially edentulous arch impressions. It contributes valuable insights into optimizing clinical workflows and advancing digital dentistry practices. This review still has several limitations that need to be considered. Firstly, it is restricted to nine in vitro studies, thus limiting the analysis to this specific type of study. The authors suggest further study could enhance comprehensiveness by integrating in vivo analysis to evaluate the effectiveness of IOS and laboratory scanners in fully and partially edentulous cases. Moreover, using specific keywords in literature searches may have disregarded relevant literature. Therefore, a further study employing a systematic review methodology is needed to obtain more conclusive evidence, with our study serving as a preliminary investigation.

## Conclusion

The conclusion of this literature review is that intraoral scanners are extensively utilized and demonstrate considerable promise for edentulous impression procedures. However, morphological differences may influence the scanning process. Therefore, the choice of a digital scanner should be founded upon the practitioner’s specific needs and characteristics of each case, with a thorough evaluation of its accuracy and capability to accommodate variations in arch morphology.

## Supplementary information

PRISMA Checklist

## Data Availability

The data that support the findings of this study are available within the article.
